# Development and validation of a machine learning-based model for 90-day prognosis outcome in spontaneous intracerebral hemorrhage patients based on non-contrast computed tomography: a multicenter retrospective observational study

**DOI:** 10.1016/j.eclinm.2025.103507

**Published:** 2025-09-12

**Authors:** Lichao Wei, Biwu Wu, Tao Guo, Dewen Ru, Chen Gao, Jiayun Wu, Aimei Wu, Hong Yue, Jin Hu, Ling Wei, Zhi Geng, Kai Wang

**Affiliations:** a Department of Neurosurgery, Huashan Hospital, Shanghai Medical College, Fudan University, Shanghai, 200040, China; b National Center for Neurological Disorders, Shanghai, 200040, China; c Shanghai Key Laboratory of Brain Function and Restoration and Neural Regeneration, Shanghai, 200040, China; d Neurosurgical Institute of Fudan University, Shanghai, 200040, China; e Shanghai Clinical Medical Center of Neurosurgery, Shanghai, 200040, China; f Center for Biomedical Imaging, University of Science and Technology of China, Hefei, 230026, Anhui, China; g Department of Neurosurgery, Jinshan Hospital, Fudan University, Shanghai, China; h Department of Neurology, The First Affiliated Hospital of Anhui Medical University, Hefei, 230022, China; i Anhui Province Key Laboratory of Cognition and Neuropsychiatric Disorders, Hefei, 230022, China; j Department of Neurology, Second People's Hospital of Hefei City, The Hefei Affiliated Hospital of Anhui Medical University, Hefei, 230022, China

**Keywords:** sICH, Prognosis, Prediction model, NCCT, Machine learning

## Abstract

**Background:**

Spontaneous Intracerebral hemorrhage (sICH) is a disease with high mortality and disability. Non-contrast computed tomography (NCCT) is the most commonly used imaging method in the diagnosis and treatment of sICH. This study aimed to develop a clinically useful prediction model for the short-term prognosis of sICH patients based on NCCT features using a machine learning model.

**Methods:**

We retrospectively collected data from sICH patients from four centers in China between January 2021 and June 2024, used data from three centers as training cohort to build the model, and another single center data for external validation. The NCCT imaging features were combined with the basic clinical characteristics of sICH patients as training features for machine learning. We developed and verified the effectiveness of five models: support vector machine (SVM), logistic regression (LR), random forest (RF), eXtreme Gradient Boosting (XGboost) and Light Gradient Boosting Machine (LightGBM). The clinical feature set, NCCT imaging feature set and fusion feature set were modeled separately and externally validated. The performance of machine learning models with different features was comprehensively evaluated using ROC curves, accuracy and other related indicators. The SHapley Additive exPlanations (SHAP) diagram was used to illustrate the importance of variables in the model, and the Sequential Forward Selection (SFS) was used to screen out the core features. Finally, a convenient and practical prognosis prediction platform was built based on the core features. This study is registered with ClinicalTrials.gov (NCT06535438).

**Findings:**

A total of 1091 sICH patients from three centers were included as the training cohort, and 102 patients from a single center were included as the external validation cohort. The LightGBM model showed the best performance in predicting the short-term prognosis of sICH patients, with an area under the receiver operating characteristic curve (AUROC) of 0.813 ± 0.012. The clinical feature cohort model (AUC: 0.822, 95% CI (0.763–0.881)), the NCCT imaging feature model (AUC: 0.770, 95% CI (0.704–0.835)) and the fusion model (AUC: 0.852, 95% CI (0.797–0.906)) were developed respectively. The external validation cohort were the clinical feature model (AUC: 0.792, 95% CI (0.689–0.894)), the NCCT imaging feature model (AUC: 0.746, 95% CI (0.637–0.855)), and the fusion feature (AUC: 0.796, 95% CI (0.694–0.897). Finally, the core factors obtained through screening, including Glasgow Coma Scale (GCS) score at admission, intraventricular hemorrhage (IVH), National Institutes of Health Stroke Scale (NIHSS) score at admission, hematoma volume, mean CT value, and black hole sign were incorporated into the model to generate a publicly accessible online platform (https://surge-ustc.shinyapps.io/multi_para_sih_prognosis/).

**Interpretation:**

The prediction model based on NCCT features established by the LightGBM model has a reliable predictive effect on the short-term prognosis of sICH patients and is of great clinical convenience and practicality.

**Funding:**

Funding provided by 10.13039/501100001809National Natural Science Foundation of China (82427808, 82171382, U23A20424, 82090034 and 82371201), the Anhui Province Clinical Medical Research Transformation Special Project (202204295107020006 and 202204295107020028) and Research Fund of Anhui Institute of translational medicine (2022zhyx-B11).

**Research in context**

**Evidence before this study**

We searched PubMed for publications, published before January 31st, 2025, using the terms “spontaneous intracerebral hemorrhage” AND “prognosis outcome” AND “machine learning” AND “non-contrast computed tomography”. Our search yielded 3 results. Although there have been studies on sICH with non-contrast computed tomography (NCCT), they were based on radiomic analysis, and no prediction model based on NCCT imaging features has been established.

**Added value of this study**

In this multicenter study, we systematically and comprehensively analyzed NCCT features of spontaneous intracerebral hemorrhage (sICH) patients and evaluated five machine learning algorithms, ultimately identifying the LightGBM model as the top performer. Feature selection demonstrated that the Glasgow Coma Scale (GCS) score at admission, National Institutes of Health Stroke Scale (NIHSS) score at admission, intraventricular hemorrhage (IVH), mean CT attenuation value, and black hole sign were core predictors of 90-day functional outcomes. Based on these key factors, we developed an interactive web-based risk calculator to enable rapid early prognosis assessment. The model exhibited robust performance in external validation using an independent multicenter cohort, confirming its clinical accuracy and practical utility.

**Implications of all the available evidence**

The LightGBM model developed in this study demonstrates strong potential as a practical tool for prognostic prediction in sICH patients, enabling clinicians to efficiently and accurately assess patient outcomes. To enhance clinical accessibility, we have deployed a user-friendly online prediction platform (https://surge-ustc.shinyapps.io/multi_para_sih_prognosis/), which is freely available and designed to provide real-time, reliable prognostic support for clinical decision-making.

## Introduction

Spontaneous intracerebral hemorrhage (sICH) refers to the non-traumatic rupture of cerebral blood vessels, leading to the accumulation of blood within the brain parenchyma.^1^ Among all stroke subtypes, sICH carries the highest mortality and morbidity rates, with 30–40% of patients succumbing within the first month of onset.^2^ Among survivors, only 20% achieve functional independence, a significantly poorer outcome compared to acute ischemic stroke.^3^^,^^4^ The accurate prediction of the prognosis for sICH patients is critical for clinical decision-making, resource allocation, and communication with family members.

Currently, numerous variables are associated with the short-term prognosis of sICH, including age, hematoma volume, spot sign, and inflammatory factors.^5^, ^6^, ^7^, ^8^ However, clinicians often face difficulties in obtaining timely and accurate prognostic information due to the need for further examination of many factors that can assess sICH prognosis. Non-contrast computed tomography (NCCT) has become the preferred method for clinical diagnosis of sICH patients due to its convenience and accuracy.^9^ Various NCCT features, such as the blend sign, black hole sign, island sign, heterogeneous density sign, hypodensity sign, fluid level sign, irregular shape sign, swirl sign, and satellite sign have been proposed and widely used in the study of sICH.^10^^,^^11^ Previous studies based on NCCT features have indicated that the signals obtained through NCCT are valuable for predicting hematoma expansion and functional prognosis following sICH.^12^^,^^13^ However, these studies did not comprehensively analyze NCCT features and mainly focused on predicting hematoma expansion.^14^^,^^15^ Therefore, using NCCT features to assist clinicians in determining the prognosis of sICH has important clinical significance. Machine learning (ML), as an advanced computer-assisted data mining and analytical approach, has demonstrated superior predictive accuracy compared to conventional statistical methods. ML algorithms have been extensively applied across multiple medical domains, including hepatic diseases,^16^ traumatic brain injury,^17^ and Alzheimer's disease^18^ with studies consistently reporting excellent predictive performance. Therefore, the introduction of ML into sICH prognosis research may significantly improve prediction accuracy.

This study aims to develop a prediction platform for sICH patients based on NCCT features by comparing different machine learning models. Finally, we established a simplified and accurate prediction platform based on the most efficient machine learning model, providing a timely, convenient and practical prediction tool for the prognosis of sICH clinical work.

## Methods

### Patients

**Fig. 1:**
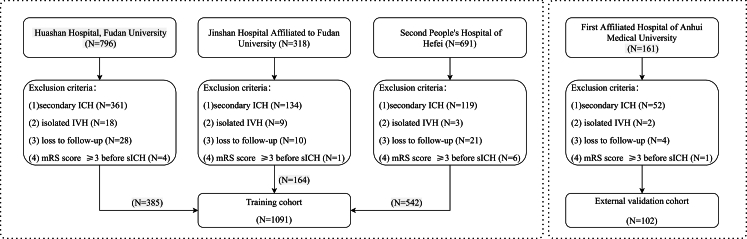
Flow diagram of the study population. (ICH: Intracerebral hemorrhage; IVH: Intraventricular hemorrhage; mRS: modified Rankin scale).

We retrospectively collected 385 sICH patients admitted to the Department of Neurosurgery of Huashan Hospital, Fudan University from January 2021 to March 2024; 164 sICH patients admitted to the Department of Neurosurgery of Jinshan Hospital Affiliated to Fudan University from March 2022 to January 2024, and 542 sICH patients admitted to the Department of Neurology of the Second People's Hospital of Hefei from January 2018 to June 2024, and composed them for training cohort. 102 sICH patients admitted to the Department of Neurology of the First Affiliated Hospital of Anhui Medical University from December 2022 to June 2024 were selected to external cohort validation the ML model (Fig. 1). This study met the sample size calculation based on the event per variable (EPV) metric, which is a widely accepted method in statistical analysis.^19^^,^^20^

The inclusion criteria included: (1) age ≥18 years; (2) meeting the diagnostic criteria for sICH, and the diagnosis was consistent with cranial NCCT scan; (3) patients had under gone initial NCCT within 48 h of symptom onset; (4) first ever acute-onset sICH.

The exclusion criteria were as follows: (1) hemorrhage due to trauma, intracranial vascular malformation, neoplasm, or any other presumed cause of secondary ICH; (2) isolated Intraventricular hemorrhage (IVH); (3) loss to follow-up; (4) patients with a modified Rankin scale (mRS) score of ≥3 before sICH.

### Data acquisition

#### Patient population

Demographic and clinicopathological variables of the enrolled patients were collected from their medical records and questionnaires and included gender, age, time from onset to CT; National Institute of Health stroke scale (NIHSS) score at admission, Glasgow Coma Scale (GCS) score at admission, Intracerebral Hemorrhage (ICH) score at admission, smoking, drinking, and basic diseases including hypertension and diabetes. Medication history included anticoagulation and antiplatelet drugs. Poor and good 90-day outcomes were defined as mRS scores of 4–6 and 0–3, respectively; which were obtained through standardized telephone interviews.

The study complies with the Declaration of Helsinki and was approved by the Ethics Committee of the Evaluation of Biomedical Research Projects of Huashan Hospital (KY2023553), Jinshan Hospital (2024-S53), Second People's Hospital of Hefei (2023-yan-018), and the First Affiliated Hospital of Anhui Medical University (2021H048), respectively. As this was a retrospective study, the requirement for informed consent was waived. This study is registered with ClinicalTrials.gov (NCT06535438).

### NCCT image acquisition and analysis

By reviewing the NCCT images of sICH patients, basic imaging information such as hemorrhage location, hematoma side, IVH, midline shift, supratentorial hemorrhage, and extension of subarachnoid hemorrhage (SAH). The long diameter, short diameter, and mean CT value were measured using the slice with the maximum haematoma size. Slice number of height represents vertical diameter of the hematoma on the coronal plane (5 mm/layer). The location of sICH was divided into lobar (involving the cerebral cortex, underlying white matter), deep (involving the thalamus, basal ganglia, brainstem or internal capsule), and infratentorial (involving the brainstem and cerebellum).^21^^,^^22^ All of those NCCT features including black hole sign, heterogeneous density sign, swirl sign, hypodensity sign, blend sign, fluid level sign, irregular shape sign, island sign, and satellite sign were evaluated and recorded by neurologists with more than 5 years of experience in the diagnosis and treatment of sICH.

### Definitions of NCCT features

The long and short diameters were defined as the maximum perpendicular diameters of the haematoma measured on the image. Slice number of height indicates the number of slices with bleeding in NCCT (slice thickness = 5 mm). Mean CT value was measured in the slice where the maximum size of hematoma. Hematoma volumes were measured using the ABC/2 method and intraventricular bleeding was not included in the volume calculations. Black hole sign,^23^ hypoattenuating area with a density difference >28 HU compared with the surrounding hematoma. No connection with surface outside the hematoma. Blend sign,^24^ relatively hypoattenuating area next to a hyperattenuating area of the hematoma, with a well-defined margin and a density difference >18 HU between the 2 areas. Heterogeneous Density sign,^25^ at least 3 foci of hypoattenuation compared with the surrounding hematoma, evaluated on the axial NCCT slice showing largest ICH area. Swirl sign,^26^ rounded, streaklike, or irregular region of hypo- or isoattenuation compared with the brain parenchyma. Hypodensity sign,^27^ any hypodense region strictly encapsulated within the hemorrhage with any shape, size, and density. Fluid level sign,^28^ presence of 1 distinct hypoattenuating area (hypodense to the brain) above and 1 hyperattenuating area (hyperdense to the brain) below a discrete straight line of separation, irrespective of its density appearance. Satellite sign,^29^ a small hematoma (diameter <10 mm) separate from the main hemorrhage in at least 1 slice and distinct from main hematoma by 1–20 mm separation. Irregular shape sign,^30^ 2 or more focal hematoma margin irregularities, joined or separate from the hematoma edge, evaluated on the axial NCCT slice showing the largest ICH area. Island sign,^31^ at least 3 scattered small hematomas all separate from the main ICH or at least 4 small hematomas some or all of which may connect with the ICH.

### Overview of machine learning models

In this study, to mitigate feature redundancy and enhance model robustness, we employed Recursive Feature Elimination (RFE) for feature selection. We evaluated five widely-used ML models: Logistic Regression (LR), Random Forest (RF), Support Vector Machine (SVM), eXtreme Gradient Boosting (XGBoost), and Light Gradient Boosting Machine (LightGBM). The reporting of this study followed the guideline of TRIPOD^32^ (Transparent Reporting of a multivariable prediction model for Individual Prognosis Or Diagnosis).

LR, a statistical model renowned for its computational simplicity and interpretability, is extensively utilized in binary classification tasks. It operates by predicting class probabilities through the Sigmoid function and optimizes the model using a logarithmic loss function. RF, an ensemble learning technique, constructs multiple decision trees and aggregates their predictions through majority voting. This method is characterized by its predictive efficiency and robustness to noise. SVM is a supervised machine learning algorithm that can be used to handle regression as well as classification problems. It enhances the classification performance and generalization ability of the model by dividing the data into decision boundaries of different categories while maximizing the margin between these boundaries and the nearest data instances. XGBoost, a gradient boosting-based ensemble algorithm, iteratively trains multiple classifiers and employs gradient descent to minimize the loss function, offering high predictive accuracy and generalization capabilities. LightGBM, another gradient boosting framework, significantly enhances computational speed and memory efficiency, making it particularly effective for handling high-dimensional data.

The dataset in the training set is divided in a ratio of 8: 2. Eight parts are allocated for model training and the remaining two parts are used to test model performance. To enhance the classifier's predictive performance and mitigate overfitting risks, we implemented a five-fold cross-validation approach combined with grid search for hyperparameter optimization. Model performance was rigorously assessed using both the training cohort and an independent external validation cohort. Evaluation metrics included the Receiver Operating Characteristic (ROC) curve, Area Under the Curve (AUC), accuracy, sensitivity, and specificity.

To increase the interpretability of the model, we utilized the SHapley Additive exPlanations (SHAP) method to elucidate the contribution of each feature to the model's predictive performance. Furthermore, in order to simplify model complexity, we utilized Sequential Forward Selection (SFS) based on the importance scores derived from SHAP to identify the core predictors. Based on these core predictors, we developed a user-friendly web-based calculator to facilitate practical application of the model.

### Sensitivity and subgroup analyses

To assess the robustness and generalizability of the model, we performed a sensitivity analysis to evaluate its stability and a subgroup analysis to examine its applicability across different patient populations. Specifically, we examined the impact of multiple imputation by chained equations (MICE) and complete case analysis on model performance. The iteration value of multiple imputation is 10 and the estimator is BayesianRidge.

Subgroup analyses were conducted within the internal test cohort. Stratified analyses based on surgical intervention versus conservative treatment were used to evaluate the model's predictive performance across clinically relevant subgroups.

### Data analysis

Data analysis and visualization were performed using R (version 4.3.1), Python (version 3.10.10), Scikit-learn (version 1.2.2), and Shiny (version 0.5.1). We performed multiple imputation and filled missing values for continuous and categorical variables separately. Categorical variables were evaluated using chi-square test or Fisher's exact test, and the results are expressed as percentages. Continuous variables that followed a normal distribution were expressed as mean ± standard deviation and examined using t-test; non-normal variables were expressed as median (interquartile range) using the Mann–Whitney U–test. Normality of continuous variables was assessed by the Kolmogorov–Smirnov test. *P*-values <0.05 (two–tailed) were considered statistically significant.

### Role of the funding source

The funder of the study had no role in study design, data collection, data analysis, data interpretation, or writing of the report.

## Results

The comparison of the baseline characteristics of the patients in the training cohort and the external validation cohort is shown in Table 1. A total of 1091 patients were included in the training development cohort, with an average age of 61.69 ± 14.46 years, 760 males (69.66%), and 331 females (30.34%). A total of 102 patients were included in the external validation cohort, with an average age of 62.17 ± 14.33 years, 74 males (72.55%), and 28 females (27.45%). There were significant differences between the training cohort and the external validation cohort in smoking (*P* < 0.001), hematoma volume (*P* < 0.001), NIHSS score at admission (*P* < 0.001), mean CT value of hematoma (*P* < 0.001), short diameter (*P* < 0.001), hematoma side (*P* = 0.004), island sign (*P* = 0.002), swirl sign (*P* < 0.001), hypodensity sign (*P* < 0.001), SAH (*P* < 0.001) and outcome (*P =* 0.031). Table 2 shows the comparative analysis of patients with good and poor outcomes in the training cohort, sex (*P* = 0.035), NIHSS score at admission (*P* < 0.001), GCS score at admission (*P* < 0.001), hematoma volume (*P* < 0.001), long diameter (*P* < 0.001), short diameter (*P* < 0.001), slice number of height (*P* < 0.001), hematoma location (*P* = 0.01), IVH (*P* < 0.001), island sign (*P* < 0.001), satellite sign (*P* < 0.001), fluid level sign (*P* < 0.001), black hole sign (*P* < 0.001), irregular shape sign (*P* < 0.001), swirl sign (*P* < 0.001), hypodensity sign (*P* < 0.001), heterogeneous density sign (*P* < 0.001), midline shift (*P* < 0.001) and SAH (*P* = 0.003).

**Table 1:** Baseline data and scale information on participants.

Characteristics	Train cohort	External validation cohort	*P*-value
N	1091	102	
**Demographic and clinical characteristics**			
Age (years)	61.69 ± 14.46	62.17 ± 14.33	0.752
Sex			0.543
Male	760 (69.66%)	74 (72.55%)	
Female	331 (30.34%)	28 (27.45%)	
Smoking			0.044
No	954 (87.44%)	82 (80.39%)	
Yes	137 (12.56%)	20 (19.61%)	
Drinking			0.466
No	928 (85.06%)	84 (82.35%)	
Yes	163 (14.94%)	18 (17.65%)	
Hypertension			0.168
No	375 (34.37%)	42 (41.18%)	
Yes	716 (65.63%)	60 (58.82%)	
Diabetes			0.165
No	929 (85.15%)	92 (90.20%)	
Yes	162 (14.85%)	10 (9.80%)	
Antiplatelet			0.929
No	981 (89.92%)	92 (90.20%)	
Yes	110 (10.08%)	10 (9.80%)	
Anticoagulation			0.138
No	1033 (94.68%)	100 (98.04%)	
Yes	58 (5.32%)	2 (1.96%)	
Time from onset to CT (hour)	4.00 (3.00–8.00)	6.00 (4.00–10.00)	<0.001
NIHSS score at admission	9.00 (4.00–16.00)	8.00 (2.00–13.00)	0.027
GCS score at admission	13.00 (8.00–15.00)	12.00 (10.00–14.00)	0.529
ICH score at admission	1.00 (1.00–2.00)	1.00 (0.00–2.00)	0.119
**CT characteristics**			
Hematoma volume (ml)	13.00 (5.00–34.00)	23.00 (10.25–38.00)	0.009
Mean hematoma CT value (Hu)	61.00 (57.00–64.00)	50.23 (45.02–57.67)	<0.001
Long diameter (mm)	34.00 (21.00–50.00)	34.00 (23.00–40.75)	0.118
Short diameter (mm)	24.00 (16.00–34.00)	18.00 (12.00–24.00)	<0.001
Slice number of height (5 mm/layer)	6.00 (5.00–8.00)	6.00 (5.00–8.00)	0.190
Hematoma side			0.004
Left	610 (55.91%)	42 (41.18%)	
Right	481 (44.09%)	60 (58.82%)	
Hemorrhage location			0.126
Lobar	336 (30.80%)	24 (23.53%)	
Deep	755 (69.20%)	78 (76.47%)	
Infratentorial hemorrhage			0.755
No	962 (88.18%)	91 (89.22%)	
Yes	129 (11.82%)	11 (10.78%)	
IVH			0.138
No	712 (65.26%)	74 (72.55%)	
Yes	379 (34.74%)	28 (27.45%)	
Island sign			0.002
No	786 (72.04%)	88 (86.27%)	
Yes	305 (27.96%)	14 (13.73%)	
Satellite sign			0.984
No	910 (83.41%)	85 (83.33%)	
Yes	181 (16.59%)	17 (16.67%)	
Fluid level sign			0.182
No	1038 (95.14%)	100 (98.04%)	
Yes	53 (4.86%)	2 (1.96%)	
Black hole sign			0.761
No	932 (85.43%)	86 (84.31%)	
Yes	159 (14.57%)	16 (15.69%)	
Irregular shape sign			0.585
No	839 (76.90%)	76 (74.51%)	
Yes	252 (23.10%)	26 (25.49%)	
Swirl sign			<0.001
No	897 (82.22%)	100 (98.04%)	
Yes	194 (17.78%)	2 (1.96%)	
Blend sign			0.020
No	818 (74.98%)	87 (85.29%)	
Yes	273 (25.02%)	15 (14.71%)	
Hypodensity sign			<0.001
No	709 (64.99%)	96 (94.12%)	
Yes	382 (35.01%)	6 (5.88%)	
Heterogeneous density sign			0.149
No	1007 (92.30%)	90 (88.24%)	
Yes	84 (7.70%)	12 (11.76%)	
Midline shift			0.050
<5 mm	955 (87.53%)	96 (94.12%)	
≥5 mm	136 (12.47%)	6 (5.88%)	
SAH			<0.001
No	1038 (95.14%)	85 (83.33%)	
Yes	53 (4.86%)	17 (16.67%)	
Outcome			0.031
Good	629 (57.65%)	70 (68.63%)	
Poor	462 (42.35%)	32 (31.37%)	

GCS: Glasgow Coma Scale; NIHSS: National Institute of Health stroke scale; ICH: Intracerebral hemorrhage; IVH: Intraventricular hemorrhage; SAH: Subarachnoid Hemorrhage; Hu: Hounsfield unit; CT: Computed tomography.

**Table 2:** Baseline characteristics of ICH patients in the Training cohort.

Characteristics	Good	Poor	*P*-value
N	629	462	
**Demographic and clinical characteristics**			
Age (years)	61.57 ± 14.04	61.87 ± 15.02	0.735
Sex			0.035
Male	454 (72.18%)	306 (66.23%)	
Female	175 (27.82%)	156 (33.77%)	
Smoking			0.581
No	553 (87.92%)	401 (86.80%)	
Yes	76 (12.08%)	61 (13.20%)	
Drinking			0.388
No	530 (84.26%)	398 (86.15%)	
Yes	99 (15.74%)	64 (13.85%)	
Hypertension			0.536
No	221 (35.14%)	154 (33.33%)	
Yes	408 (64.86%)	308 (66.67%)	
Diabetes			0.190
No	528 (83.94%)	401 (86.80%)	
Yes	101 (16.06%)	61 (13.20%)	
Antiplatelet			0.191
No	572 (90.94%)	409 (88.53%)	
Yes	57 (9.06%)	53 (11.47%)	
Anticoagulation			0.213
No	591 (93.96%)	442 (95.67%)	
Yes	38 (6.04%)	20 (4.33%)	
Time from onset to CT (hour)	4.00 (3.00–9.00)	4.00 (2.00–8.00)	0.646
NIHSS score at admission	6.00 (2.00–10.00)	14.00 (8.00–20.00)	<0.001
GCS score at admission	14.00 (13.00–15.00)	9.00 (6.00–13.00)	<0.001
ICH score at admission	1.00 (0.00–2.00)	2.00 (1.00–3.00)	<0.001
**CT characteristics**			
Hematoma volume (ml)	9.00 (3.00–22.00)	23.00 (9.00–53.00)	<0.001
Mean hematoma CT value (Hu)	60.00 (56.00–65.00)	61.00 (57.00–64.00)	0.703
Long diameter (mm)	30.00 (18.00–41.00)	42.00 (28.00–60.00)	<0.001
Short diameter (mm)	20.00 (14.00–30.00)	29.00 (20.00–40.00)	<0.001
Slice number of height (5 mm/layer)	6.00 (4.00–8.00)	8.00 (6.00–10.00)	<0.001
Hematoma side			0.284
Left	343 (54.53%)	267 (57.79%)	
Right	286 (45.47%)	195 (42.21%)	
Hemorrhage location			0.010
Lobar	213 (33.86%)	123 (26.62%)	
Deep	416 (66.14%)	339 (73.38%)	
Supratentoria hemorrhage			0.286
No	80 (12.72%)	49 (10.61%)	
Yes	549 (87.28%)	413 (89.39%)	
IVH			<0.001
No	487 (77.42%)	225 (48.70%)	
Yes	142 (22.58%)	237 (51.30%)	
Island sign			<0.001
No	514 (81.72%)	272 (58.87%)	
Yes	115 (18.28%)	190 (41.13%)	
Satellite sign			<0.001
No	563 (89.51%)	347 (75.11%)	
Yes	66 (10.49%)	115 (24.89%)	
Fluid level sign			<0.001
No	613 (97.46%)	425 (91.99%)	
Yes	16 (2.54%)	37 (8.01%)	
Black hole sign			<0.001
No	586 (93.16%)	346 (74.89%)	
Yes	43 (6.84%)	116 (25.11%)	
Irregular shape sign			<0.001
No	546 (86.80%)	293 (63.42%)	
Yes	83 (13.20%)	169 (36.58%)	
Swirl sign			<0.001
No	547 (86.96%)	350 (75.76%)	
Yes	82 (13.04%)	112 (24.24%)	
Blend sign			<0.001
No	517 (82.19%)	301 (65.15%)	
Yes	112 (17.81%)	161 (34.85%)	
Hypodensity sign			<0.001
No	466 (74.09%)	243 (52.60%)	
Yes	163 (25.91%)	219 (47.40%)	
Heterogeneous density sign			<0.001
No	607 (96.50%)	400 (86.58%)	
Yes	22 (3.50%)	62 (13.42%)	
Midline shift			<0.001
<5 mm	598 (95.07%)	357 (77.27%)	
≥5 mm	31 (4.93%)	105 (22.73%)	
SAH			0.003
No	609 (96.82%)	429 (92.86%)	
Yes	20 (3.18%)	33 (7.14%)	

GCS: Glasgow Coma Scale; NIHSS: National Institute of Health stroke scale; ICH: Intracerebral hemorrhage; IVH: Intraventricular hemorrhage; SAH: Subarachnoid Hemorrhage; Hu: Hounsfield unit; CT: Computed tomography.

**Fig. 2:**
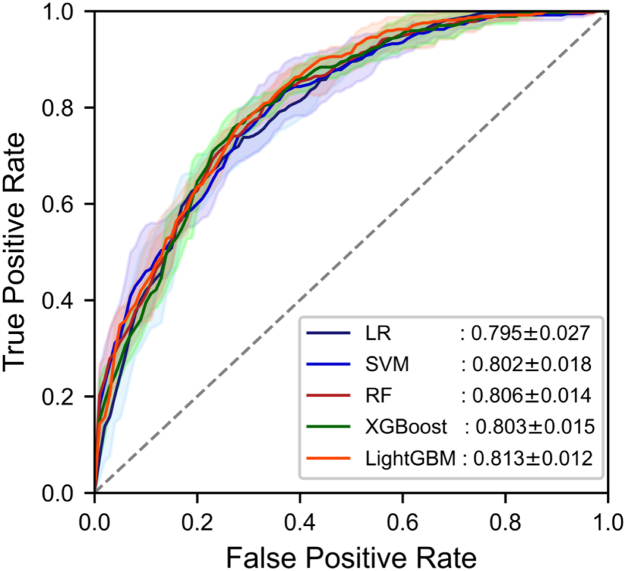
The average AUC performance of five machine learning models subjected to fivefold cross-validation.

**Fig. 3:**
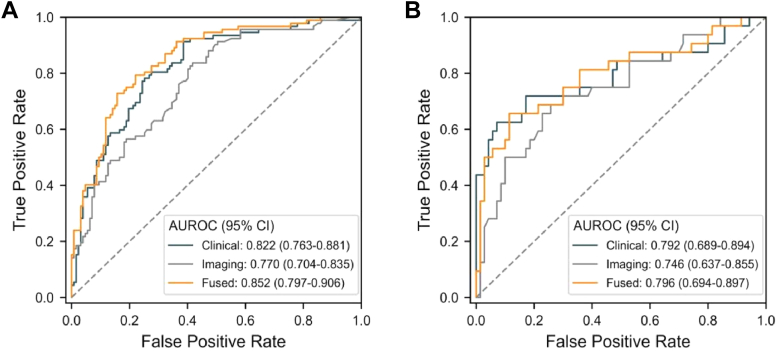
ROC curve analysis of the LightGBM model based on clinical features, NCCT features, and fusion features to predict the short-term prognosis of sICH patients (A. Internal test; B. External validation).

Perform five-fold cross validation on the five machine learning models in the training cohort and obtain the average prediction performance of each model, LR (0.795 ± 0.027), SVM (0.802 ± 0.018), RF (0.806 ± 0.014), XGBoost (0.803 ± 0.015), LightGBM (0.813 ± 0.012) (Fig. 2). Table 3 shows the comparison of common performance indicators between different machine learning prediction models. Based on these results, we selected the LightGBM model (AUC = 0.813) as our prediction model. In the internal test, clinical features (AUC: 0.822, 95% CI (0.763–0.881)), NCCT features (AUC: 0.770, 95% CI (0.704–0.835)) and the fusion of the two (AUC: 0.852, 95% CI (0.797–0.906)) (Fig. 3A). Although the AUCs of the three models decreased slightly in the external validation, including clinical features (AUC: 0.792, 95% CI (0.689–0.894)), NCCT features (AUC: 0.746, 95% CI (0.637–0.855)), and the fusion of the two (AUC: 0.796, 95% CI (0.694–0.897)), the fusion feature model still had the best detection performance (Fig. 3B) (Table 4).

**Table 3:** Comparative analysis of the performance outcomes across 5 machine learning models.

Model	F1 Score	Accuracy	Recall	Precision	AUC	Specificity	Sensitivity
LR	0.664	0.728	0.632	0.701	0.795	0.799	0.632
SVM	0.639	0.718	0.586	0.704	0.802	0.815	0.586
RF	0.683	0.737	0.668	0.700	0.806	0.789	0.668
XGBoost	0.693	0.741	0.689	0.698	0.803	0.779	0.689
LightGBM	0.683	0.733	0.681	0.689	0.813	0.771	0.681

LR: Logistic regression; RF: Random forest; XGBoost: Extreme gradient boosting; LightGBM: Light gradient boosting machine; SVM: Support vector machine; AUC: Area under the curve.

**Table 4:** Performance of GBM models with different feature combinations on the internal test and external validation.

	Clinical features internal test	Image features internal test	Fused features internal test	Clinical features external validation	Image features external validation	Fused features external validation
F1 Score	0.670	0.613	0.741	0.667	0.590	0.615
Accuracy	0.735	0.694	0.776	0.784	0.686	0.706
Recall	0.641	0.576	0.761	0.688	0.719	0.750
Precision	0.702	0.654	0.722	0.647	0.500	0.522
AUC(95%)	0.822	0.770	0.852	0.792	0.746	0.796
Sensitivity	0.641	0.576	0.761	0.688	0.719	0.750
Specificity	0.803	0.780	0.787	0.829	0.671	0.686

AUC: Area under the curve.

**Fig. 4:**
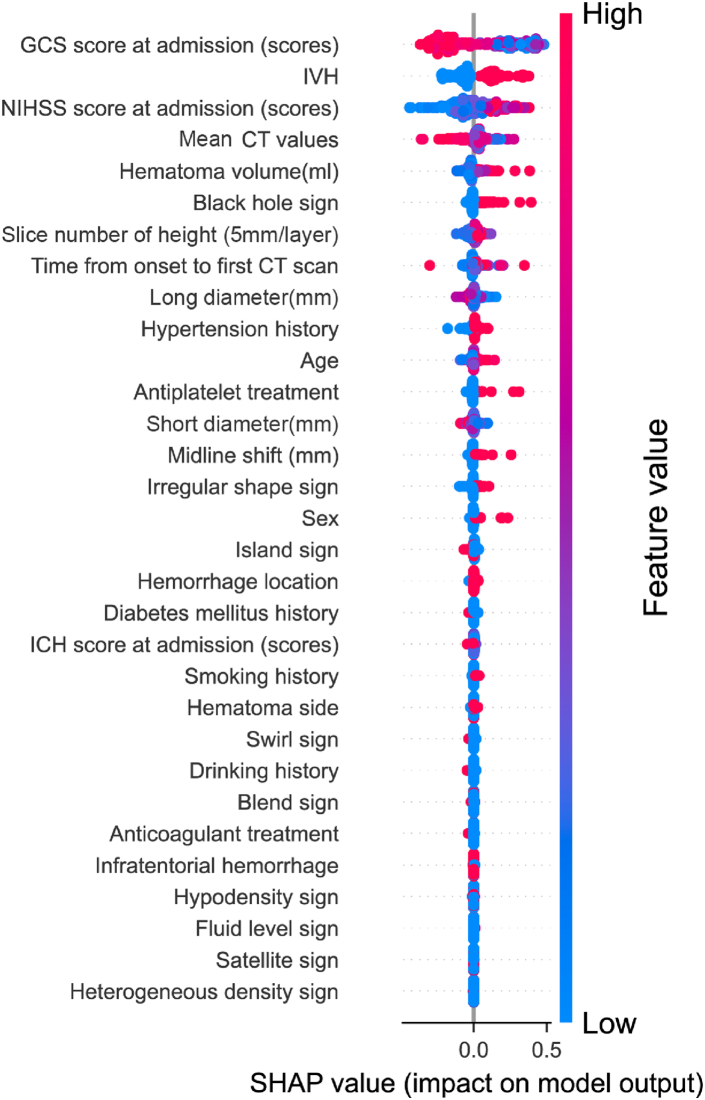
SHAP analyses of the LightGBM model for predicting poor prognosis of sICH patients. (GCS: Glasgow Coma Scale; NIHSS: National Institute of Health stroke scale; ICH: Intracerebral hemorrhage; IVH: Intraventricular hemorrhage; CT: Computed tomograph).

**Fig. 5:**
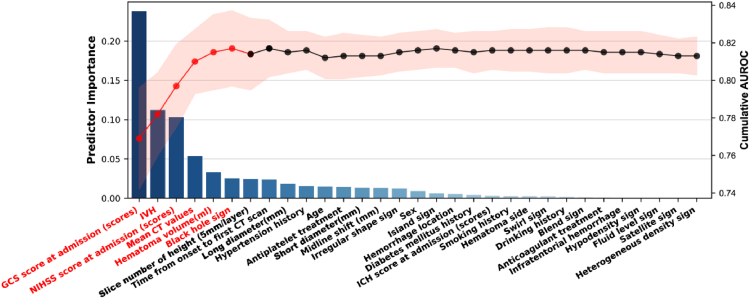
Comparison of the performance derived from LightGBM model constructed with various variable combinations based on variable importance. (GCS: Glasgow Coma Scale; NIHSS: National Institute of Health stroke scale; ICH: Intracerebral hemorrhage; IVH: Intraventricular hemorrhage; CT: Computed tomograph).

**Fig. 6:**
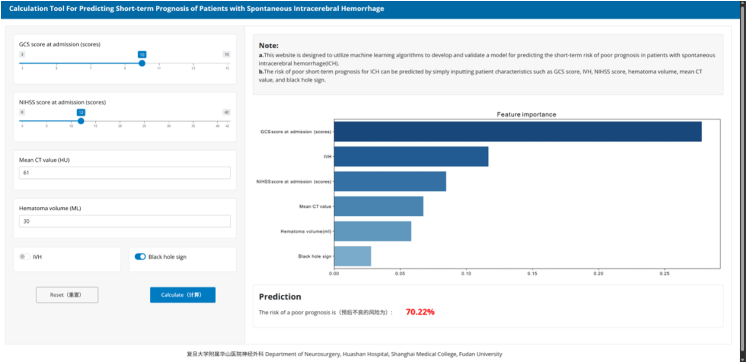
A web-based calculator for predicting short-term prognosis in sICH patients.

The SHAP graph was employed to visualize the impact of predictive variables on the model's outcomes, delineating the contributions of multiple factors including GCS score at admission, NIHSS score at admission, IVH, mean CT value, hematoma volume, black hole sign, island sign, and swirl sign to the predictive results (Fig. 4). Furthermore, We used the sequential forward selection (SFS) method to further simplify the model; and generated the combined AUC and variable importance line plots using the prediction data derived from the variable importance and combination in the LightGBM model. As showed in Fig. 5, by integrating GCS score at admission, IVH, NIHSS score at admission, mean CT value, hematoma volume, and black hole sign, the predictive capability of the model is optimized and streamlined. Utilizing these six critical indicators, we developed a web-based calculator to facilitate the individualized prognostic risk assessment for sICH patients, enhancing both accessibility and convenience (https://surge-ustc.shinyapps.io/multi_para_sih_prognosis/; Fig. 6).

As shown in Supplementary Table S1 and Supplementary Figure S1, the model demonstrated good performance in both subgroups, with AUC values of 0.804 (95% CI, 0.710–0.899) for the surgical intervention group and 0.845 (95% CI, 0.763–0.926) for the conservative treatment group.

In the internal test cohort, the AUC of the model using MICE was 0.796 (95% CI: 0.694–0.897), while the complete case analysis with an AUC of 0.827 (95% CI: 0.696–0.957) (Supplementary Table S2 and Supplementary Figure S2A). In the external validation cohort, the AUC values were 0.852 (95% CI: 0.797–0.906) for the MICE and 0.828 (95% CI: 0.766–0.891) for the complete case analysis (Supplementary Table S3 and Supplementary Figure S2B). These results indicate that the two methods achieved relatively similar AUC values and maintained high model performance overall.

## Discussion

This study built a machine-learning-based web tool for predicting the prognosis of sICH by integrating commonly used NCCT features and basic clinical features. A series of data-driven selection methods were used, and it was determined that GCS score at admission, NIHSS score at admission, hematoma volume, IVH, mean CT value, and black hole sign were the core predictors of prognosis. By comparing different prediction models, LightGBM model was finnally selected as the prediction model with an AUC of 0.813, indicating a high predictive performance. When tested with an external validation data set, the model also showed excellent accuracy and reliability. The clinical prognosis prediction tool we proposed is extremely convenient and practical in the clinical environment, and has made a huge contribution to early clinical decision-making.

ML methods have become an important tool to improve the performance of disease risk prediction models.^33^, ^34^, ^35^ A study by Qi et al.^36^ have shown that the LightGBM model has excellent ability in predicting the occurrence of cardiovascular diseases-cancer comorbidity. In our study, the LightGBM model used the simplest combination of variables to achieve the best prediction performance, which was significantly better than other models. At the same time, in the external validation cohort, the AUC of the LightGBM model reached 0.796, indicating that the LightGBM model has strong generalization ability, confirming its applicability in clinical prediction of short-term prognosis of sICH patients.

The NIHSS score is widely used to assess the severity of stroke in clinical trials of acute ischemic stroke.^37^ In recent years, clinicians have also paid more and more attention to the evaluation of NIHSS scores for sICH patients.^38^^,^^39^ A retrospective analysis of 417 sICH patients showed that the NIHSS score played an important role in predicting the short-term prognosis of sICH.^40^ This is consistent with our research results. Similarly, the GCS score at admission, as a commonly used scoring scale for evaluating sICH patients, has also been confirmed in a large number of studies.^41^, ^42^, ^43^ These results show that the more severe the neurological damage in sICH patients at the onset of the disease, the worse their recovery. How to reduce early neurological damage and early neurological recovery treatment is still the focus of sICH treatment research.

Hematoma volume is one of the important factors for predicting poor prognosis of sICH, but the cutoff value of hematoma volume has not been clearly defined.^44^ Our study also showed that an increase in hematoma volume significantly increased the risk of poor prognosis of sICH, which is consistent with most current models for predicting the prognosis of sICH, indicating that hematoma volume is a stable predictor. Our study not only compared the effect of hematoma volume on the prognosis of sICH, but also analyzed the length, width and height of hematoma volume as a separate factor. Unfortunately, these variables have not been screened as core variables for predicting poor prognosis, which may be due to the hematoma morphology of sICH is usually irregular, and the length, width and height may not accurately reflect the true spatial distribution of the hematoma. Previous studies have reported that the presence of IVH is a strong and independent predictor of functional prognosis after sICH, but the specific mechanism is unclear.^38^^,^^42^^,^^45^, ^46^, ^47^ Some studies believe that it may be the direct compression effect of IVH on periventricular structures, especially on the brainstem, and IVH may also cause cerebrospinal fluid circulation disorders, increased intracranial pressure and spread of inflammatory reactions, causing damage to brain tissue.^48^^,^^49^ The results of this study are consistent with most previous studies that found that the presence of IVH can affect the functional prognosis of sICH. However, a study have expressed a different view, believing that IVH is not a factor affecting the prognosis of sICH and should not be used as a categorical variable in the study of sICH.^50^

The black hole sign, blend sign, fluid level sign, hypodensity sign, and heterogeneous density sign are all distinct imaging manifestations that arise from density differences observed on NCCT. The irregular shape sign, island sign, and satellite sign represent the morphological variations of hematomas as depicted on NCCT. In 2016, Li Qi^23^ first proposed that the black hole sign could serve as a predictor for hematoma expansion. Subsequently, in 2017, Li Qi^51^ demonstrated that the presence of the black hole sign on NCCT correlates with a poor functional prognosis at 3 months following sICH. Various NCCT signs, including the blend sign, fluid level sign, heterogeneous density sign, hypodensity sign, irregular shape sign, island sign, and satellite sign, have been associated with poor prognosis in sICH patients.^11^^,^^27^^,^^28^^,^^31^^,^^52^^,^^53^ Our study confirms significant differences in these features between the poor prognosis group and the good prognosis group. Nevertheless, in our pursuit of an optimal predictive model, only the black hole sign was screened as a crucial variable. The importance score of the black hole sign was notably higher than that of other NCCT features in our study. This could be attributed to the requirement for a clear CT value to define a black hole sign, enabling a more objective prediction of poor prognosis in sICH, with less susceptibility to subjective influences. Moreover, as most NCCT signs are based on density variations, lower mean CT values suggest NCCT features with potentially greater density heterogeneity, making them significant variables in the prediction model. A study by Chu demonstrated that the minimal CT attenuation value independently predicts poor outcome in sICH patients.^54^

Several prediction models have been developed to assess the prognosis of sICH. However, some of these models have shown limited predictive accuracy, potentially undermining their reliability.^41^ In contrast, more accurate models often require extensive evaluation of predictive variables, limiting their clinical usefulness. For example, models incorporating point signs require refinement of CT angiography (CTA) examination, while radiomics predicting model require further data processing.^55^^,^^56^ Moreover, some models lack external validation, hindering a comprehensive assessment of their predictive capacity.^57^ Our study addressed these limitations by ensuring that the sample size ensured the robustness of the model and using external centers for external validation of the model. Additionally, feature selection based on NCCT improved the clinical applicability of the model.

The strength of this study is that the prediction performance of different machine learning models was established and compared using multi-center, large sample size data. Comparison between the clinical features prediction model, NCCT features prediction model, and fusion model showed that combining clinical features with NCCT features can significantly improve the prediction performance of the model. In addition, for further application, we transformed the complex prediction algorithm into an intuitive and easy-to-use clinical tool through a web-based calculator. This tool has the potential to assist clinicians in risk stratification, personalized treatment plans, optimized resource allocation, and improved communication with patients and their families.

Certainly, our study has some limitations. Firstly, its retrospective design may introduce bias, highlighting the need for prospective studies for higher-quality evidence. Secondly, despite a multicenter approach, the predominantly Asian study population warrants validation of the model's generalizability to other ethnicities. Thirdly, although we developed a high-performing prognostic model based on NCCT images, the absence of advanced imaging parameters such as MRI and CTA remains an important limitation of this study. MRI can provide additional information regarding perihematomal edema,^58^ post-hemorrhagic microstructural alterations (diffusion tensor imaging),^59^ and brain tissue metabolism,^60^ all of which have been shown to be closely associated with neurological outcomes. Similarly, CTA can detect abnormal vascular signs such as the spot sign,^61^ leakage sign,^62^ and iodine sign,^63^ which are well-established predictors of hematoma expansion and poor prognosis. These advanced imaging modalities not only offer deeper insight into the dynamic evolution of the hematoma, bleeding mechanisms, and secondary injury processes, but also demonstrate high sensitivity and specificity. Incorporating such parameters may contribute to the development of a more physiologically grounded and biologically informed predictive model. However, in routine clinical practice, the use of MRI and CTA is often limited by factors such as patient instability, cost, and narrow imaging time windows, resulting in relatively low utilization rates—particularly in emergency and resource-limited settings. Therefore, in this study, we deliberately chose NCCT as the primary imaging modality due to its widespread availability, rapid acquisition, and compatibility with early clinical workflows. Future studies could consider integrating multimodal imaging data to explore the additive prognostic value of multidimensional imaging biomarkers in sICH, which may further improve model accuracy and clinical interpretability.

Fourthly, this study did not incorporate treatment strategies into the predictive model, which represents an important limitation. Currently, the management of sICH is broadly categorized into two main approaches: medical and surgical treatment. Medical treatment protocols have been continuously refined in recent years. Emerging clinical evidence suggests that comprehensive early interventions—including the early intensive lowering of systolic blood pressure, strict glucose control, antipyrexia treatment, and rapid reversal of warfarin-related anticoagulation—can significantly improve functional outcomes.^64^ Incorporating these therapeutic factors into prognostic models may further enhance predictive accuracy. Regarding surgical treatment, although various techniques are available—including decompressive craniectomy, endoscopic surgery, stereotactic aspiration, and external ventricular drainage—recent high-quality studies indicate that only minimally invasive surgery has demonstrated consistent benefits in improving outcomes in selected sICH patients.^65^, ^66^, ^67^ The effectiveness of other surgical approaches remains controversial, with considerable heterogeneity in terms of surgical modality, timing of intervention, and intraoperative strategy, all of which add complexity to modeling. Although we performed subgroup analyses comparing surgical versus medical patients and observed that the model maintained stable performance across treatment categories, future studies should aim to systematically collect detailed variables such as treatment modality, timing, and intensity. This would allow the development of a dynamic, treatment-informed prediction model and enable exploration of the model's value in guiding personalized therapeutic decision-making.

Finally, laboratory test results were not included in this study. White blood cell count,^68^ hemoglobin,^69^ and D-dimer^70^ have been associated with prognosis in sICH. However, their acquisition requires blood sampling and lab processing, resulting in delays, and their values may be influenced by comorbidities unrelated to the hemorrhage itself, reducing their objectivity in emergency care. This study aims to build a rapid and intuitive prognostic assessment tool based on NCCT features. Therefore, we excluded laboratory variables to prioritize feasibility and early clinical utility. Nonetheless, prospective studies should aim to build multimodal, dynamic models by incorporating imaging, treatment, and laboratory data to enhance prediction performance and personalization.

This study screened clinical characteristics and NCCT features, and established a prediction model using the LightGBM model, integrating the NIHSS score on admission, GCS score on admission, IVH, mean CT value, hematoma volume, and black hole sign as predictive factors. This model exhibits a dependable predictive capacity for the short-term prognosis of sICH patients. Additionally, it offers clinicians a convenient and intuitive tool for forecasting the short-term prognosis of sICH patients in early clinical practice.

## Contributors

Conception and design (LCW, DRR, AMW, ZG), data collection (LCW, BWW, DRR, HY, ZG), data analysis (LCW, TG, ZG, KW), drafting (LCW, CG), helping with drafting (JYW, AMW, HY, LW, JH), draft revision (LCW, ZG, KW), approval of final version (LCW, BWW, TG, DRR, JYM, AMW, HY, JH,LW, ZG, KW). Dr Lichao Wei and Zhi Geng had accessed and verified all the data in the study and take responsibility for the integrity of the data and the accuracy of the data analysis.

## Data sharing statement

Data could be available from corresponding author.

## Declaration of interests

The authors report no conflicts of interest in this work.
