# Supplementary material for: Development and validation of a machine learning-based model for 90-day prognosis outcome in spontaneous intracerebral hemorrhage patients based on non-contrast computed tomography: a multicenter retrospective observational study
Source: eClinicalMedicine. 2025 Sep 12;88:103507. doi: 10.1016/j.eclinm.2025.103507 (PMC12572785; doi:10.1016/j.eclinm.2025.103507)

**Supplementary Table 1**. Subgroup analysis results of the internal test.

| Subgroup | F1 Score | Accuracy | Recall | Precision | AUC | Specificity | Sensitivity |
| --- | --- | --- | --- | --- | --- | --- | --- |
| Conservative treatment | 0.860 | 0.800 | 0.925 | 0.803 | 0.804 | 0.925 | 0.556 |
| Surgical intervention | 0.560 | 0.763 | 0.538 | 0.583 | 0.845 | 0.538 | 0.850 |

**Supplementary Table 2.** Comparison of model performance using MICE imputation versus complete case analysis in internal test cohort.

| Model | F1 Score | Accuracy | Recall | Precision | AUC | Specificity | Sensitivity |
| --- | --- | --- | --- | --- | --- | --- | --- |
| MICE | 0.741 | 0.776 | 0.761 | 0.722 | 0.852 | 0.761 | 0.787 |
| complete case | 0.735 | 0.737 | 0.735 | 0.735 | 0.828 | 0.735 | 0.738 |

**Supplementary Table 3**. Comparison of model performance using MICE imputation versus complete case analysis in external validation.

| Model | F1 Score | Accuracy | Recall | Precision | AUC | Specificity | Sensitivity |
| --- | --- | --- | --- | --- | --- | --- | --- |
| MICE | 0.625 | 0.714 | 0.882 | 0.517 | 0.827 | 0.882 | 0.641 |
| complete case | 0.615 | 0.706 | 0.750 | 0.522 | 0.796 | 0.750 | 0.686 |

**Supplementary Table 4**. Variance inflation factor (VIF) for the selected features.

| Feature | VIF |
| --- | --- |
| Hematoma volume(ml) | 6.675 |
| GCS score at admission (scores) | 4.451 |
| ICH score at admission (scores) | 3.759 |
| NIHSS score at admission (scores) | 3.468 |
| The length of the largest level of the hematoma (mm) | 3.451 |
| The width of the largest level of the hematoma (mm) | 2.747 |
| Number of slices of the hematoma on CT scan(5mm/ per layer) | 2.474 |
| Island sign | 1.922 |
| Irregular shape sign | 1.773 |
| Midline shift (mm) | 1.691 |
| IVH | 1.663 |
| Blend sign | 1.624 |
| Hypodensity sign | 1.569 |
| Drinking history | 1.544 |
| Smoking history | 1.540 |
| Heterogeneous density sign | 1.536 |
| Black hole sign | 1.412 |
| Swirl sign | 1.397 |
| Satellite sign | 1.371 |
| Infratentorial hemorrhage | 1.346 |
| Hemorrhage location | 1.239 |
| Age | 1.170 |
| Sex | 1.145 |
| Fluid level sign | 1.134 |
| CT values | 1.103 |
| Time from onset to first CT scan | 1.094 |
| Hypertension history | 1.072 |
| Hematoma side | 1.065 |
| Diabetes mellitus history | 1.059 |
| Anticoagulant treatment | 1.032 |
| Antiplatelet treatment | 1.027 |

**Supplementary Table 5.** Variance inflation factors(VIF) for the 6 features included in the web-based calculator.

| Feature | VIF |
| --- | --- |
| GCS score at admission (scores) | 3.545 |
| NIHSS score at admission (scores) | 3.108 |
| Hematoma volume(ml) | 1.552 |
| Black hole sign | 1.250 |
| IVH | 1.165 |
| CT values | 1.007 |

**Supplementary Table 6.** AUC DeLong test results for the LightGBM model with different features.

| Model 1 | Model 2 | AUC 1 | AUC 2 | *P*-value |
| --- | --- | --- | --- | --- |
| Internal test |  |  |  |  |
| Fusion features | Clinical features | 0.852 | 0.822 | 0.022 |
| Fusion features | Imaging features | 0.852 | 0.770 | 0.004 |

AUC: Area Under the ROC Curve; LightGBM: Light gradient boosting machine.

**Supplementary Figure 1**. ROC curve of the LightGBM model for surgical intervention subgroup analysis in internal test.

**
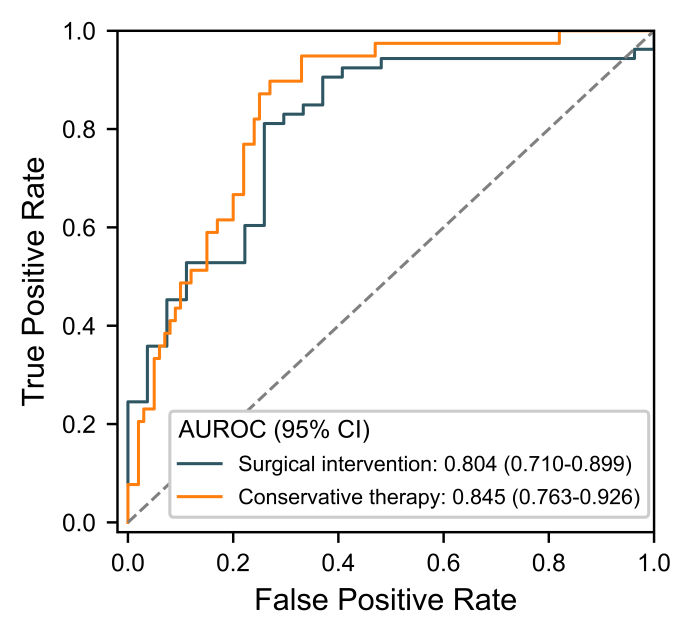
**

**Supplementary Figure 2**. ROC curve of the LightGBM model based on MICE and comeplete case analysis. (A. Internal test; B. External validation).


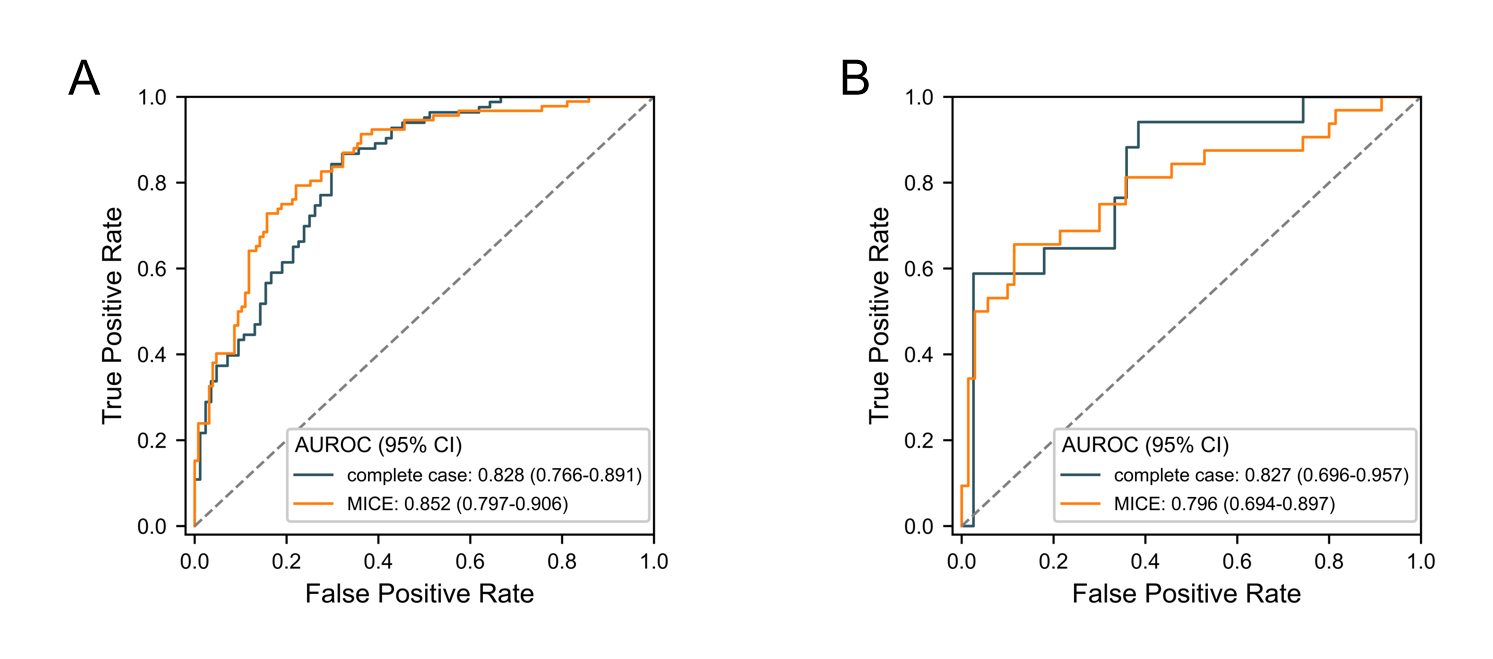

Supplement: Supplementary Figures and Tables [file mmc1.docx]
